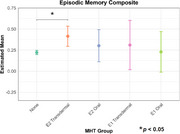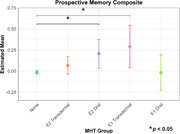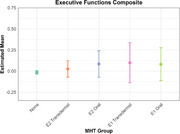# One Size Does Not Fit All: Menopause Hormone Therapy Type and Route of Administration Influences Cognitive Health

**DOI:** 10.1002/alz70861_108141

**Published:** 2025-12-23

**Authors:** Laura Gravelsins, Tanvi A Puri, Madeline Wood Alexander, Andrew J McGovern, Paula Duarte‐Guterman, Jennifer S Rabin, Kelly J Murphy, Liisa AM Galea

**Affiliations:** ^1^ Centre for Addiction and Mental Health, Campbell Family Mental Health Research Institute, Toronto, ON Canada; ^2^ University of British Columbia, Vancouver, BC Canada; ^3^ Hurvitz Brain Sciences Program, Sunnybrook Research Institute, Toronto, ON Canada; ^4^ Brock University, St. Catharines, ON Canada; ^5^ Sunnybrook Research Institute, Toronto, ON Canada; ^6^ Baycrest, Toronto, ON Canada; ^7^ Springboard Clinic, Toronto, ON Canada; ^8^ University of Toronto, Toronto, ON Canada

## Abstract

**Background:**

Menopause, characterized by a significant decline in ovarian hormones, represents a key transition point in the aging trajectory (Gravelsins & Galea, in press). Use of menopausal hormone therapy (MHT) has been associated with reduced risk of Alzheimer’s disease dementia as well as benefits for cognition and the brain (Kim et al., 2021). However, other studies have found MHT to have no benefit, or negative effects, on dementia risk, cognition, and the brain (Espeland et al., 2013). These inconsistencies may be explained by a failure to account for MHT formulation, MHT administration route, and cognitive domain assessed. Estradiol (E2), the most potent of the estrogens, has the greatest binding affinity to estrogen receptors (ER). Comparatively, estrone (E1) has significantly lower binding affinity. Oral E2 is largely converted to E1 via first‐pass metabolism. In contrast, transdermal E2 largely circumvents this conversion, resulting in higher circulating E2 (Gleason et al., 2005). The cognitive domains of executive functions and episodic memory may be differentially sensitive to MHT, as they rely on different brain regions. Few studies have examined the effects of E2‐ and E1‐based MHT based on administration route on different cognitive domains.

**Method:**

Using baseline data in 4,776 mostly healthy postmenopausal females from the Canadian Longitudinal Study of Aging, we examined the influence of E2‐ and E1‐based MHT on performance in three cognitive domains: episodic memory, prospective memory, and executive functions.

**Result:**

We found that transdermal E2 was associated with higher episodic memory scores (*p*=0.0084, Cohen’s *d*=0.228), whereas oral E2 and transdermal E1 were associated with higher prospective memory scores (E2: *p*=0.0353, Cohen’s *d*=0.296; E1: *p*=0.0353, Cohen’s *d*=0.409), compared never use of MHT. Neither MHT formulation nor route of administration influenced the executive functions composite.

**Conclusion:**

These results underscore the differential effects of E2‐ and E1‐based MHT depending on route of administration and cognitive domain tested. Notably, these results may reflect dose‐dependent effects of estrogens; episodic memory may benefit from greater ER activation than prospective memory. This work clarifies the mixed MHT literature and may inform emerging precision medicine approaches for cognitive aging in females.